# An analytical toolkit for polyploid willow discrimination

**DOI:** 10.1038/srep37702

**Published:** 2016-12-09

**Authors:** Wei Guo, Jing Hou, Tongming Yin, Yingnan Chen

**Affiliations:** 1 Co-Innovation Center for Sustainable Forestry in Southern China, College of Forestry, Nanjing Forestry University, Nanjing, China

## Abstract

Polyploid breeding is an important means for creating elite willow cultivars, and therefore provokes an active demand for discriminating the ploidy levels of natural willow stands. In this study, we established an analytical toolkit for polyploid willow identification by combining molecular markers and flow cytometry (FCM). A total of 10 single-copy fully informative SSRs were chosen for marker-aided selection based on a segregation test with a full-sib willow pedigree and a mutability test with a collection of natural willow stands. Aided by these molecular markers, we performed polyploid selection in two tree species and two shrub species of the genus *Salix*. The ploidy levels of the investigated samples were further examined using a flow cytometer. It was previously shown that results from marker-aided selection were consistent with those from FCM measurements. Based on ploidy level assessment in different willow species, it was found that tree willows were dominantly tetraploid, whereas shrub willows were most frequently diploid. With this analytical toolkit, polyploids can be rapidly screened from a large number of natural stands; thereafter, the exact ploidy levels of the polyploid candidates can be efficiently confirmed by FCM. This analytical toolkit will greatly enhance polyploid breeding programs for willows.

Polyploids are widespread in plants, especially in angiosperms. It is estimated that approximately 30–80% of angiosperms are polyploid^1^,^2^,^3^. Polyploidy has long been recognized as a major force driving higher plant evolution and diversification^4^,^5^. With the expansion of genome size, physiological and developmental characteristics of organisms are also substantially modified, which results in phenotype changes that may increase their adaptation capacity^6^. In general, polyploidisation increases leaf and flower size, stomatal density, and cell size^7^, and this is collectively referred to as the gigas effect^8^. This property has been ubiquitously applied in breeding programs for agricultural and ornamental plants. Polyploids have great breeding value, because they can have higher yields, and greater tolerance to biotic and abiotic stresses^9^,^10^.

The genus *Salix*, a member of the Salicaceae family, and consists of 350–500 species in forms of trees, sub-trees, and shrubs^11^,^12^. Members of this genus are divided into four subgenera: *Salix, Longifoliae, Vetrix*, and *Chamaetia*^11^. Many willow species can achieve high biomass yields through short growth cycles with low agrochemical inputs^13^; thus, they are considered promising sources for bioenergy production^12^,^14^. In addition, *Salix* is one of the few woody plants with a large number of polyploid taxa^15^. The basic chromosome number of this genus is 19, and the ploidy level ranges from diploid (2n = 38) to dodecaploid (12n = 228)^16^,^17^. Around 40% of *Salix* species are polyploids, and many species exhibit more than one ploidy level^17^. For example, *S. fragilis*, which belongs to the subgenus *Salix*, is mainly tetraploid (4n = 76), but diploid (2n = 38) and hexaploid (6n = 114) are also observed^18^. It has been suggested that palaeopolyploidisation occurred several times in *Salix*^19^,^20^. Recently, sequencing the *S. suchowensis* genome, which is a member of subgenus *Vetrix*, revealed that the willow genome contained the most recent whole-genome duplication event that took place around 58 million years ago^21^.

Breeding and genetic improvement of willows through controlled pollination and hybridisation has led to the production of many novel cultivars suitable for bioenergy production^12^,^22^. These novel hybrids display significant variation in biomass production. Significant difference has been observed between ploidy level and growth in some willow species^23^. In general, triploid willows are more vigorous and produce higher yield than their diploid and tetraploid parents^24^,^25^, and it has been demonstrated that the triploid and tetraploid willows possessed lower lignin content than the diploid genotypes^23^. Considering the significant effects of ploidy level on growth and wood composition, ploidy determination is critical for polyploid willow breeding programs^26^.

Traditionally, assessing the ploidy level of plants is conducted by counting the number of chromosomes at metaphase during cell division^26^,^27^. However, cytological counting of willow chromosome numbers is very difficult because of their high chromosome numbers and small chromosome size^28^,^29^. Nowadays, flow cytometry (FCM) is widely adopted for determining the ploidy level of organisms^30^. Many studies have demonstrated FCM efficiency for ploidy level estimation for different plant species, including *Salix* species^29^. However, for FCM analysis, sample preparation is complicated and laborious because of plant cell wall rigidity^30^; thus, it is not suitable for large-scale analyses.

By contrast, molecular markers provide an efficient, rapid, and cost effective means to analyse a large number of samples. Using fully informative molecular markers, we can identify polyploid candidates based on the observed allele numbers, and the candidates can then be confirmed by FCM analysis. This combined approach was shown to be very efficient for discriminating polyploids in natural poplar stands^31^. Additionally, an effective method for screening polyploids is also highly desirable for willow breeding programs. In this study, we developed an analytical tool to detect polyploids from natural willow stands by combining marker-aided selection and FCM analysis.

## Materials and Methods

### Plant Materials

We selected two tree willow species (*S. babylonica* and *S. matsudana*, subgenus *Salix*) and two shrub willow species (*S. suchowensis* and *S. integra*, subgenus *Vetrix*) for the tests in this study. Cuttings were collected from 12 different stands for each species from the willow germplasm nursery maintained at Chenwei Forestry Farm in Jiangsu Province, China. The collected cuttings were then propagated in the Nanjing Forestry University campus greenhouse. Young leaves were collected from each individual, and DNA was extracted using the CTAB method, as described by Murray and Thompson^32^.

### SSR Primer Development and Amplification Test

Based on the *S. suchowensis* genome sequences^21^, we developed 192 SSR primer pairs (Table S1), and these primers were synthesised by Jerry Bio Ltd, Shanghai, China. To test their success in PCR amplification, we randomly selected a DNA template from each of the four willow species. PCRs were carried out as described by Tuskan *et al*.^33^, and amplification products were visualized on GelRed^TM^-stained (Biotium, Hayward, CA, USA) 1% agarose gels. The primers that were successfully amplified in all four willow species were subjected to the following tests.

### Selection of Single-copy Fully Informative SSRs

The SSR primers that succeeded in PCR amplification were further examined with an F_1_ full-sib pedigree of *S. suchowensis*, as described by Hou *et al*.^34^. In this study, the two mapping parents and six progeny were employed to examine segregation of the amplified alleles. The mapping parents of this pedigree were diploid; thus, a single-copy fully informative marker should generate two alternate alleles in each of the parents. In the mapping pedigree, each progeny will separately inherit one of the alternate alleles from the mother and the father. Based on the segregation of parental alleles in the progeny, we can unambiguously identify the single-copy fully informative SSRs. In detail, microsatellites that genotype as AB in the mother, and genotype as BC or CD in the father were determined to be single-copy fully informative markers, where A, B, C and D refer to the alternate alleles at a particular SSR locus. When analysing a natural stand with a single-copy fully informative SSR, the individual could be a polyploid candidate if more than two alleles are generated.

### Variability Test and Marker-Aided Selection of Polyploid Willows

When examined with a single-copy fully informative SSR, only heterozygous loci can be visualized as distinctable alternate alleles. The heterozygosity of an SSR locus depends on its variabitility. The higher the variability of an SSR marker, the higher efficiency it has for identifying polyploids. Therefore, the variability of all the detected single-copy fully informative SSRs were further surveyed by genotyping the aforementioned 12 *S. suchowensis* stands. The PCR amplicons were analysed on an ABI 3730 sequencer (Applied Biosystems, Foster City, CA, USA), and alleles were called by ABI GeneMapper software (Version 3.7). Polymorphism information content (PIC) associated with each SSR marker was calculated by the formula described in Kong *et al*.^31^.

Finally, the highly variable single-copy fully informative SSRs were selected and used for marker-aided selection of polyploid willows. Ploidy discrimination was performed on a total of 48 willow stands, as described in Plant Materials.

### Polyploid Willow Verification by FCM

To verify ploidy levels, all samples were analysed on a BD Influx flow cytometer (Becton Dickinson Biosciences, San Jose, CA, USA). The instrument was equipped with an air-cooled argon-ion laser tuned at 15 mW and operated at 488 nm. For each calibration, the instrument was optimized using Sphero^TM^ rainbow calibration particles (Spherotech, Lake Forest, IL, USA). Sample preparation was performed following a modified protocol according to Doležel *et al*.^35^. About 100 mg fresh leaves were rapidly chopped with a sharp razor blade in 2 mL ice-cold Galbraith’s buffer^36^. Then, 1 mL suspension was filtered through a 40-μm nylon mesh to remove debris. The filtered suspension was incubated under dark conditions in 50 μg/mL propidium iodide (Sigma, St Louis, MO, USA) and 50 μg/mL RNase (Takara, Dalian, China) at 4 °C for 30 min. Fluorescence emitted from the DNA-binding propidium iodide was collected with a 670-nm dichroic long-pass filter. Measurements were called and analysed using BD FACS™ (Version 1.0.0.650, Becton Dickinson Biosciences, San Jose, CA, USA). Three repeats were performed for each sample, and the sequenced diploid *S. suchowensis*^21^ was employed as the reference sample.

Ploidy level was calculated according to the following formula: 
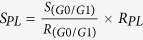
; where 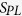 is the ploidy level of the measured sample, 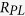 is the ploidy level of the reference sample, 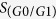 is the mean position of the G_0_/G_1_ peak for the measured sample (G_0_/G_1_, cells in G_0_ or G_1_ phase), and 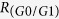 is the mean position of the G_0_/G_1_ peak for the reference sample.

## Results

### SSR Primer Amplification and Selection of Single-copy Fully Informative SSRs

**Figure 1:**
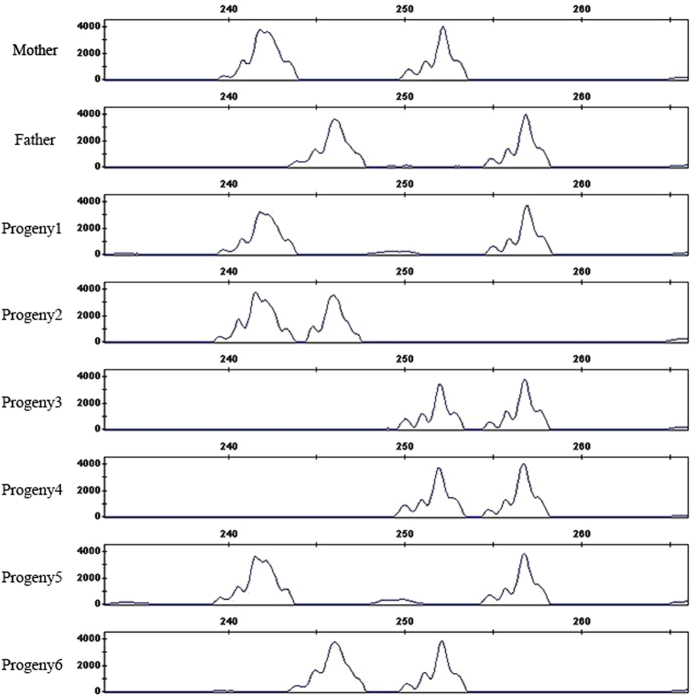
Segregation of alleles generated by the primer WSSR_100 in the F_1_ full-sib pedigree of *Salix suchowensis.* Note: the genotype of the mother is AB, and the genotype of the father is CD.

By examining the PCR products through agarose gel electrophoresis, it was found that 174 primers (90.6% of all primers) were successfully amplified across the four tested willow species. Subsequently, the successful primers were amplified against DNA templates from the full-sib pedigree of *S. suchowensis* to determine their copy number and informativeness. Figure 1 showed representative electropherograms generated by primer WSSR_100, the genotype of the mother was AB, and the genotype of the father was CD, the possible genotypes of the progeny were AC, AD, BC, or BD. With such a test, we could exclude multi-copy SSRs and SSRs that generated null alleles. Finally, we obtained 11 single-copy fully informative SSR primers that amplified distinct alleles that can be easily recorded.

### Variability Test

The selected single-copy fully informative SSR primers were further subjected to a variability test by genotyping against the aforementioned 12 *S. suchowensis* individuals. Based on the genotypes of these individuals, Ssu_17 and Ssu_38 were identified as ramets of the same clone. In addition, five SSR primers were found to amplify three alleles in sample Ssu_90, which indicates that Ssu_90 might be a triploid candidate. When different ploidy samples are mixed, allele frequencies cannot be estimated precisely because of some marker genotypes being phenotypically indistinguishable. Thus, statistics for the variability test were performed by excluding samples Ssu_17 and Ssu_90. Genotyping profiles of the 11 primers produced allele numbers that varied from 3 to 6, with an average of 4.6. The sizes of the amplicons were from 168 bp to 406 bp. PIC values ranged from 0.34 to 0.79, with an average of 0.63. Normally, an SSR with a PIC value > 0.5 is considered a highly variable marker^37^. Finally, 10 SSRs that generated distinct and easily recordable alleles in natural stands and had PIC values greater than 0.5 were chosen as diagnostic markers for discriminating the ploidy levels of natural willow stands (Table 1).

### Polyploid Candidate Identification

**Figure 2:**
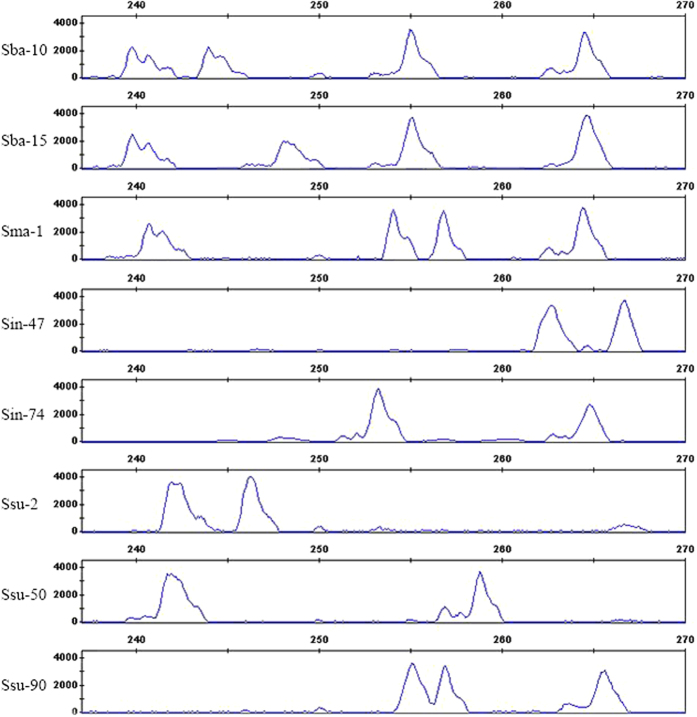
A demonstration of segregation of alleles generated by the single-copy fully informative primer WSSR_100 in the four willow species.

Twelve individuals from each species were genotyped with the selected diagnostic markers. Clustering analysis of the genotyping data showed that some of the samples were clonal ramets, e.g. Sba_1, Sba_2, and Sba_4 of *S. babylonica*; and Sma_7 and Sma_11 of *S. matsudana* (Figure S1). In the genotyping data matrix (Table S2), the majority of genotyping data points were formulated by one or two alleles. Besides, 35 genotyping data points are in formulation of three alleles, and 32 in four alleles (Table S2). The genotyping profile of primer WSSR_100 is shown in Fig. 2 as an example. In a diploid plant, a single-copy fully informative SSR should amplify at most two alleles at a particular locus. Thus, samples that contained genotyping data that revealed three or four alleles were inferred to be polyploidy candidates. For example, out of the 10 diagnostic SSR primers, four (WSSR_33, WSSR_89, WSSR_100, and WSSR_173) amplified four alleles at most in sample Sin_270 of *S. integra*, which indicates that Sin_270 might be a tetraploid candidate; four primers (WSSR_34, WSSR_91, WSSR_94, and WSSR_100) amplified three alleles at most in sample Ssu_90 of *S. suchowensis*; thus, Ssu_90 might be a triploid candidate; three primers (WSSR_88, WSSR_94, and WSSR_100) amplified two alleles at most in sample Sma_7 of *S. matsudana*; and no primers amplified more than two alleles, which indicates that Sma_7 might be a diploid candidate.

Based on the revealed maximum allele number for each sample in the genotyping data matrix (Table S2), eight *S. matsudana* stands were inferred to be tetraploid candidates, and the remaining *S. matsudana* were diploid candidates; for *S. babylonica*, all 12 stands were identified as tetraploid candidates; for *S. integra*, only one stand was inferred to be a tetraploid candidate, and the others were diploid candidates; and for *S. suchowensis*, one stand was inferred to be a triploid candidate, and the others were diploid candidates. Therefore, the majority of tree willow stands were tetraploid candidates. On the contrary, diploid candidates dominated the shrub willow stands.

### Ploidy Level Verification by FCM

To verify the ploidy levels revealed by marker-aided selection, the examined samples were further measured using a BD Influx flow cytometer. The instrument gain was set with the G_0_/G_1_ peak approximately on channel 10,000 by taking the sequenced individual of *S. suchowensis* as reference, and instrument settings were kept constant throughout the measurements. In each run, at least 5,000 particles for each sample were measured. Quality of the peaks was evaluated by the coefficient of variation (CV). Generally, measurements with CV values smaller than 5% are considered reliable^35^,^38^,^39^. In our measurements, CV values ranged from 2.61–4.97% (mean 4.47%).

**Figure 3:**
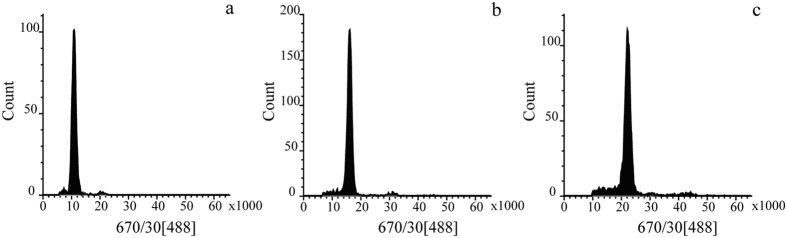
Flow cytometric histograms of three different ploidy levels of *Salix* (**a**) Indicates the flow cytometric histograms of diploid reference sample Ssu_2. (**b**) Indicates the flow cytometric histograms of triploid sample Ssu_90. (**c**) Represents the flow cytometric histograms of tetraploid sample Sba_15.

Ratios for the mean G_0_/G_1_ peak positions of the samples over that of the reference ranged from 0.9 to 2.09 (Table 2), and fluctuated slightly either around 1.0, 1.5, or 2.0, which indicated that the measured samples were diploid, triploid, or tetraploid (Fig. 3). Based on FCM measurements, eight *S. matsudana* stands were confirmed to be tetraploids, and the remaining *S. matsudana* were diploids; all 12 *S. babylonica* stands were tetraploids; only one *S. integra* stand was tetraploid, and the others were diploid; and one *S. suchowensis* stand was triploid, whereas the others were diploids. The results obtained in this study confirmed that ploidy measurements by FCM (Table 2) were consistent with those inferred from the genotyping data matrix (Table S2). Therefore, with the 10 diagnostic SSRs, we obtained reliable estimates for the ploidy levels of stands from different willow species.

## Discussion

Natural polyploids more commonly occur in pteridophyte and flowering plants than in animals^40^,^41^,^42^. Many agricultural plants, such as wheat, banana, and some crops in the genus *Brassica*, are polyploids. It is well known that vegetation growth varies with ploidy level, and polyploid forms tend to grow better than the genets in diploid form for many plants^9^,^10^. Thus, polyploid breeding has long been a useful strategy to complement conventional diploid breeding. *Salix* is one of the few woody genera with a wide ploidy spectrum, among which diploid and tetraploid are the most common vegetative forms. In Salicaceae, triploids have been generally known to display improved vigor and form; for example, a series of studies on *P. tremula* indicated that triploids exhibited the best vegetation growth among different ploidy levels^43^,^44^,^45^,^46^. Recently, Serapiglia *et al*. demonstrated that triploid shrub willows produced higher biomass yield than their diploid and tetraploid parents^23^. As in many plants, polyploid breeding is also a highly desirable means for breeding elite willow cultivars. Thus, there is an active demand to develop rapid and reliable analytical toolkit to discriminate the ploidy levels of natural willow stands.

Polyploids can be identified based on morphological and physiological characteristics with limited accuracy. Alternatively, we can directly identify polyploids by examining chromosome number under microscopes or by measuring DNA content with a flow cytometer. However, such methods are laborious and time-consuming, especially when dealing with a large number of samples. Compare to these conventional strategies, molecular markers provide a highly efficient and reliable means to conduct large-scale selection of polyploids from natural stands. The efficiency of marker-aided selection for polyploids depends on the heterozygosity of amplified loci; in many cases, the exact ploidy levels cannot be determined merely based on molecular markers. However, marker-aided selection enables us to narrow down the polyploids to a small number of candidates, and thus greatly improves the efficiency of FCM analysis. Kong *et al*.^31^ demonstrated the power of the combining marker-aided selection and FCM for screening polyploid poplars. In this study, we established an associated analytic toolkit for detecting polyploid willows, and our results showed the feasibility and reliability of this toolkit for practical selection.

In this study, development and screening of molecular markers were mainly conducted using *S. suchowensis* DNA. In addition to this species, the selected markers were also successful in ploidy discrimination for three other willow species that represented both tree and shrub willows. The power of diagnostic markers for polyploid identification is highly correlated with mutability of the amplified loci. Normally, SSR markers are highly transferable among species, and may even be transferable across *taxa* of genera^47^. In Salicaceae, some SSRs were transferable across the genera *Salix* and *Populus*^33^,^48^. However, there is a tradeoff between transferability and variability of SSR markers^49^,^50^. Therefore, the usability of these diagnostic SSRs needs to be cautiously tested when these SSRs are applied for detection of polyploids in more diverged willow species.

Genotyping data revealed that some of the examined stands with different accession numbers were actually clonal ramets, especially in *S. matsudana* and *S. babylonica*. Germplasm records showed that samples of these two tree willow species were originally collected from Xuanwu Lake Park and Zijin Mountain in Nanjing of China. Willows in these scenic areas were artificially planted, and many of them might be propagated by cuttings from the same genotype. By contrast, the two shrub willow species, *S. integra* and *S. suchowensis*, were originally collected from Maoer Mountain in Heilongjiang Province and Xinyi in Jiangsu Province of China, respectively. The two shrub willow species are mainly maintained through naturally dispersed seeds. Accordingly, clonal ramets were relatively rare in the tested samples of these two shrub willows.

Ploidy level survey indicated that stands of the two examined shrub willow species mainly existed in diploid form. On the contrary, most of the investigated tree willow stands were tetraploids. The dominant ploidy level varied between tree and shrub willow species, which was also observed in previous studies. By microscopically examining chromosome number, Suda and Argus^17^ explored the ploidy levels of 21 willow species, including one tree willow species, four sub-tree/shrub willow species, and 16 shrub willow species. Among these, the tree willow species (*S. alba*) was identified as tetraploid; two of the sub-tree/shrub willow species (*S. amygdaloides* and *S. arbusculoides*) were diploid; and the other two sub-tree/shrub willow species (*S. discolor* and *S. scouleriana*) were tetraploid. Regarding the 16 shrub willows, ploidy level varied dramatically: nine shrub species (*S. brachycarpa, S. candida, S. exigua, S. interior, S. lutea, S. monticola, S. myrtillifolia, S. petiolaris*, and *S. silicicola*) were diploid; one (*S. subcoerulea*) was triploid; two species (*S. humilis* and *S. pellita*) and one hybrid (*S. athabascensis* × *pedicellaris*) were tetraploid; and the other three shrub species had more than one level of polyploidy, such as triploid/tetraploid in *S. planifolia*, hexaploid/octaploid in *S. glauca*, and decaploid/dodecaploid in *S. maccalliana*. Thibault^29^ found that ploidy levels of 10 willow species and five hybrids were examined by measuring DNA content with a flow cytometer. Among these, two species (*S. alba* and *S. fragilis*) and a hybrid (*S.* ×*chrysocoma*) were tree willows, and they all appeared to be tetraploid. Regarding the shrub willows, five species (*S. caprea, S. elaeagnos, S. purpurea, S. triandra*, and *S. pyrenaica*) and two hybrids (*S.* ×*rubra* and *S.* ×*quercifolia*) were diploid; two hybrids (*S.* ×*mollissima* and *S.* ×*stipularis*) were triploid; two species (*S. atrocinerea* and *S. cinerea*) were tetraploid; and one species, *S. viminalis*, was observed to have four diploids and one tetraploid.

In summary, willow species in tree form are mainly tetraploid, and only occasionally diploid. By contrast, ploidy levels of shrub willow species have been shown to vary greatly, with diploid predominating the different ploidy levels. Although the dominant ploidy level differs between tree and shrub willows, the plant form of willows should not be triggered by the ploidy level of their genomes. The genetic mechanism underlying the plant form of willows needs to be explored at a deeper molecular level. Nevertheless, we established an analytic toolkit capable of large-scale discrimination of natural willow stand ploidy levels, which is highly desirable for facilitating willow polyploid breeding programs.

## Additional Information

**How to cite this article**: Guo, W. *et al*. An analytical toolkit for polyploid willow discrimination. *Sci. Rep.* **6**, 37702; doi: 10.1038/srep37702 (2016).

**Publisher's note:** Springer Nature remains neutral with regard to jurisdictional claims in published maps and institutional affiliations.

## Supplementary Material

Supplementary Information

## Figures and Tables

**Table 1:** Ten SSR primers selected to detect polyploid willows.

Primer name	Forward primer (5′-3′)	Reverse primer (5′-3′)	Parental genotypes^a^	PIC value
WSSR_11	TTTATAATGGCCATGAGCTT	TCACTAGGTCCTGGAACATC	AB × BC	0.54
WSSR_33	GTCATTTACAGGTCTGGCAT	GAGGTTGATGTTTGGTAAGG	AB × BC	0.71
WSSR_34	CCCTAGAAAGGAAGGACAAT	CAATGAGTTTGTGATGGTGA	AB × BC	0.62
WSSR_88	CACAAATCTTATTGGAAAAC	TTACTACTGATGCTGTTC	AB × CD	0.76
WSSR_89	TTGGCAGTTATGTCTCCA	AGTTTGTCCAAGTGTCCC	AB × BC	0.57
WSSR_91	CATCGTGCCCAGTAAGGA	ACATAGGAAGCGGGTGGT	AB × CD	0.54
WSSR_94	ACAAGGCATCAAAGTAGCA	CTCCAGGAGATCCAAGACG	AB × BC	0.68
WSSR_100	GCAAAAGCCAAAAGGAGA	AACCAGCAGAGGAAAGTG	AB × CD	0.79
WSSR_124	TGCTCTGAAAGATCTACGGT	AACCACATTGATTCTTCCAC	AB × CD	0.67
WSSR_173	TTATTGCTGGAAAGGTTG	TTCGTGTCTTTAGGGTCT	AB × BC	0.69

^a^Genotypes were determined by the type of segregation of alleles generated by the primers in the F_1_ full-sib pedigree of *Salix suchowensis*.

**Table 2:** Ploidy level estimates of four *Salix* species by flow cytometry.

Accession No.	Species	G_0_/G_1_ mean	Ratio^a^	Ploidy level	CV (%)
Sba_1, Sba_2, Sba_4	*S. babylonica*	22182	2.07	4×	3.85
Sba_5, Sba_7, Sba_9	*S. babylonica*	21500	2.00	4×	3.87
Sba_10, Sba_11	*S. babylonica*	22341	2.08	4×	3.4
Sba_13, Sba_14	*S. babylonica*	21213	1.98	4×	4.04
Sba_15, Sba_17	*S. babylonica*	21731	2.02	4×	3.95
Sma_2	*S. matsudana*	9931	0.92	2×	4.49
Sma_1, Sma_3, Sma_5	*S. matsudana*	22470	2.09	4×	3.9
Sma_6, Sma_9	*S. matsudana*	20529	1.91	4×	4.93
Sma_7, Sma_11	*S. matsudana*	11826	1.10	2×	4.78
Sma_16	*S. matsudana*	11697	1.09	2×	4.68
Sma_18, Sma_21, Sma_26	*S. matsudana*	22366	2.08	4×	2.61
Sin_47	*S. integra*	10920	1.02	2×	4.96
Sin_74, Sin_99	*S. integra*	11457	1.07	2×	4.94
Sin_134	*S. integra*	10900	1.01	2×	4.94
Sin_137, Sin_221	*S. integra*	9947	0.93	2×	4.85
Sin_270	*S. integra*	22557	2.10	4×	4.35
Sin_491	*S. integra*	10686	1.00	2×	4.88
Sin_551	*S. integra*	10345	0.96	2×	4.77
Sin_578	*S. integra*	11401	1.06	2×	4.86
Sin_579	*S. integra*	11275	1.05	2×	4.74
Sin_608	*S. integra*	10354	0.96	2×	4.93
Ssu_1	*S. suchowensis*	10441	0.97	2×	4.97
Ssu_2 (Reference)	*S. suchowensis*	10739	1.00	2×	4.81
Ssu_17, Ssu_38	*S. suchowensis*	10878	1.01	2×	4.38
Ssu_47	*S. suchowensis*	11133	1.04	2×	4.56
Ssu_50	*S. suchowensis*	10502	0.98	2×	4.9
Ssu_69	*S. suchowensis*	11349	1.06	2×	3.61
Ssu_90	*S. suchowensis*	15753	1.47	3×	4.12
Ssu_99	*S. suchowensis*	11681	1.09	2×	4.57
Ssu_101	*S. suchowensis*	9712	0.90	2×	4.92
Ssu_107	*S. suchowensis*	11103	1.03	2×	4.95
Ssu_120	*S. suchowensis*	11380	1.06	2×	4.49

^a^Ratio was calculated by dividing the mean position of the peak (G_0_/G_1_) for the measured sample by the mean position of the peak for the diploid *S. suchowensis*, which was 10739.
