# Supplementary material for: An analytical toolkit for polyploid willow discrimination
Source: Sci Rep. 2016 Dec 9;6:37702. doi: 10.1038/srep37702 (PMC5146657; doi:10.1038/srep37702)
Supplement: Supplementary Information [file srep37702-s1.pdf]

An analytical toolkit for polyploid willow discrimination

Wei Guo, Jing Hou, Tongming Yin, Yingnan Chen*

Co-Innovation Center for Sustainable Forestry in Southern China, College of Forestry, Nanjing Forestry University, Nanjing, China

Running title: Marker aided selection of polyploid willow

*Author for Correspondence:

Yingnan Chen

College of Forestry, Nanjing Forestry University, Nanjing, China

Tel: 01186-25-85428165

Fax: 01186-25-85427165

Email:chenyingnan@njfu.edu.cn

Supplementary materials

Table S1 192 SSR primer pairs developed from *Salix suchowensis* genome

Table S2 Allele number at each locus generated by 10 SSR primers in the four willow species

Fig. S1 Clustering analysis of the genotyping data generated by the selected diagnostic markers in the 48 individuals from four willow species

Table S1 192 SSR primer pairs developed from *Salix suchowensis* genome

Primer name	Forward primer (5'-3')	Reverse primer (5'-3')	Scaffold
WSSR_1	AATTTCTGGAGGCTCTTCTT	GCCTTAAACAAATCCACATC	scaffold1
WSSR_2	ACGGTGAAGTTTGTGATTTC	ACCATTCTCCACCCTTAGAT	scaffold1
WSSR_3	CAACCACAATCGGAATTTAT	CAAGAGGAACTTGAGTGGAC	scaffold1
WSSR_4	ATTCCCAGCAACTTGTTAAA	TACATTTCGAGTTCATTTCG	scaffold1
WSSR_5	TTGAATGAGAATGAAATTGAAA	AGAAATGTCATTATTTGGTGATT	scaffold1
WSSR_6	CAGAATTGGAAGAGGAAGTG	TGCATACGAGTGAACATCAT	scaffold2
WSSR_7	ACTTCCATCACCATTACTCG	AACCTCTCCTCTTCCGATAC	scaffold3
WSSR_8	TGACAATTCAATGGTATGGA	GTATTCGGATGGCTTTACTG	scaffold4
WSSR_9	AAACACCATCTCTTCACACC	GCAAAGGAACAATCAATAGC	scaffold5
WSSR_10	TTCTCCGATTCACTATTGCT	GTACCACTACTCGCTCCAAC	scaffold5
WSSR_11	TTTATAATGGCCATGAGCTT	TCACTAGGTCCTGGAACATC	scaffold5
WSSR_12	CTTGAGTTCATTCTGTGCAA	AAGACTGCAGATAGGTCGAA	scaffold6
WSSR_13	AAACCTGATGAATGTATGGC	GCAAGCTCGCTTTAATTTAG	scaffold6
WSSR_14	TTTATCTATCAAATTTGGTCCT	TTTCCGATGTTTGGTAGTGT	scaffold6
WSSR_15	CAGCAGCTCATGAGTTATCA	CAGATGGTGTCATTTCTCCT	scaffold6
WSSR_16	TTGGTCGATATCGGTAATTC	CCTCTTAGGAAGGAAGTGGT	scaffold6
WSSR_17	TCAATTATTTCACACCAGCA	CTCCTGGTTTCTGTGTCATT	scaffold6
WSSR_18	TTTAACCAGAGGAACTGGAA	CTCCTATCGATCTGTTGAGC	scaffold7
WSSR_19	AGACATCGAACCAGGTCTAA	TTTCTTCAATTTCCATCCAC	scaffold7
WSSR_20	TTAATGGCTAAATTGGATGG	TTGACAGCTTCACACATAGG	scaffold8
WSSR_21	GCTTGAGTTTAAGGGTGTTG	CTTCAACCATGATTCCAAAT	scaffold9
WSSR_22	CCACCAGAAACTACTGCTTC	ACCAGATGTTGAAACCAAAC	scaffold9
WSSR_23	GGGAGTCTCGAAATAAGGTT	AGCTTTCGAAGTTTCTGTCA	scaffold10
WSSR_24	GAATGGGAATGAAATTGAAA	ATCAAATTTGGTCCTCAAAC	scaffold11
WSSR_25	CAGGTGAGTGTGTGTGAGAG	CACTCTCCTCCATTTCTCAG	scaffold12
WSSR_26	CTCCTCAACTCCTTCGATTA	AACAAGGCAATGTTCCTTTA	scaffold13
WSSR_27	CATGGATTTCACCTTTCTGT	TCTCCTAAGCCAGCACTTAC	scaffold14
WSSR_28	TTTGTTGTCCAATAGTGCAG	ATGGCATCCAAGTGATTAGT	scaffold15
WSSR_29	AGGTTGACTTGGCTGTTCTA	ATGATCAAGTGGACGAGTTC	scaffold15
WSSR_30	ACGTTTCAGTCTCCTGCTAA	AAGGATTTGTTGACATCAGG	scaffold16
WSSR_31	GAGGTGGACTCATACCTTGA	AAGAGTGGCAATTAGTGCAT	scaffold16
WSSR_32	GTTGTCTTCAAATCCGACAT	ACATGGAGCTCGAAGAAATA	scaffold17
WSSR_33	GTCATTTACAGGTCTGGCAT	GAGGTTGATGTTTGGTAAGG	scaffold17
WSSR_34	CCCTAGAAAGGAAGGACAAT	CAATGAGTTTGTGATGGTGA	scaffold17
WSSR_35	GTGATCAAACGAGAGAGAGC	TAATTGCAGGGTATCGATTT	scaffold19
WSSR_36	GCACTCGATCTTTGTTCTTC	GAGCATAGAGGAGCACATTC	scaffold19
WSSR_37	CAATCCTCTTTCTTCACTGG	TTAGTGCCTTGTGGAAACTT	scaffold19
WSSR_38	GGGACAGTAAACAATGGAGA	AGACCATCTATGGAGACCCT	scaffold20
WSSR_39	ATCATCCAGCAAGTAACCAC	CGCATATTTCGTACATGTTG	scaffold21
WSSR_40	ACTAAGAAGCACGAAGTGGA	ATAAACAGTGTGCAGAGGCT	scaffold21
WSSR_41	TTACCAAAGCCAAATCAAGT	TCCAGGGAGCTATCTTGTTA	scaffold21

Primer name	Forward primer (5'-3')	Reverse primer (5'-3')	Scaffold
WSSR_42	CTCCAGAATATCCACCTCCT	AAAGTAATGAAAGGGCAACA	scaffold22
WSSR_43	ATGTCTTTGTCCTTGGATTG	GAGGAGAAGAAATGGGACTT	scaffold23
WSSR_44	CCTCTGGAAGCAATACACTC	CAAGTATTTGGATTCGGAAG	scaffold23
WSSR_45	GGAACATGAAGTGTTGGACT	TCATGAGGAGGGTTTATTGT	scaffold24
WSSR_46	AAACAAACAAAGGCAATGAT	TTTAGAGTGGCTGTGGTTCT	scaffold26
WSSR_47	CATCCCAGTCACACCTTATT	TTGAGAGGCTGAAGCATATT	scaffold26
WSSR_48	GGTTGAAACTATTGGGTTGA	ACATCCTGGACTCAGAATTG	scaffold26
WSSR_49	TGTCAGCAATGAACACAGAT	ATACAATCAATGAGATGCCC	scaffold27
WSSR_50	AGAAATGTCATTATTTGCTGA	TTGAATGAGAATGAAATTGAAA	scaffold31
WSSR_51	ACTACCGCGCTTACAAATAG	AGACAACAACATCAACAGCA	scaffold31
WSSR_52	TCTGGGCTGCTTATAAGTTC	TATTGAACCACCAGGATACC	scaffold32
WSSR_53	AACACTTTCCAGAAGTTGTGA	GTTGAATGAGAATGAATTTGAA	scaffold33
WSSR_54	ATCAGCATATCCGGCTAATA	GAATAGCCCTTATCCAAGGT	scaffold33
WSSR_55	TAAGCAGCGAATATGTTTCA	TGATCTAGGTTTCCTGCAAT	scaffold35
WSSR_56	CATGAACAGACGAGACAATG	TGTTTCCACAAATCTGAACA	scaffold35
WSSR_57	TCTTGTTGGGATCTCGTTAC	CTGAACCTTAGGCTGAACAC	scaffold35
WSSR_58	ACTGTCGTTTAAGGGCAATA	TCCACTGCCTTGTAGAGATT	scaffold36
WSSR_59	CAAATATGAACACTGCGCTA	GGTGTTGATTGTGGAAGATT	scaffold38
WSSR_60	TTTAGATTCTTGGCATTGCT	TGCTCAGAGATCAAAGGATT	scaffold38
WSSR_61	GCTAAAGCTCTTGGTCTGAA	GGTTACCTGAGAAATCCACA	scaffold38
WSSR_62	TTGTATGTTTATGCTGGCAA	ATACCACCACCAAGTCCTC	scaffold39
WSSR_63	CAAATTTGATCCTCATTCTTT	TTTCAGTGTTTGGTTATGTCA	scaffold39
WSSR_64	TAATTGCTATTTGCTTTCCC	TCCAGTGTTTGGTTATGTCA	scaffold39
WSSR_65	TTAAAGGATTTGCCATGTTT	CGTTTACGACTCATCTTTCC	scaffold43
WSSR_66	TGTCAGGATTCAACTTGTGA	AGATTTGAATTAGCCTTCCC	scaffold44
WSSR_67	AACGGCAAGTTAGAAGATGA	TTAAGCCATGGTTCAGATTT	scaffold45
WSSR_68	CCTCCATCCACAGAGTAGA	CTTCATAGATTTGTTCCATTT	scaffold46
WSSR_69	TCACTACTTCTGCGATACCT	TTTCTAAACTTGTTGGGCTA	scaffold46
WSSR_70	AAGCAAGCAAAAGTCAAGAG	AGTATGCCAAGCAAGAAGAA	scaffold46
WSSR_71	AAACACCAAACAACAAATCC	GATGGTTACACCGTTATCGT	scaffold46
WSSR_72	GCTCCATTTCATTAGTCCAA	ACGAAGAAGAAGACGATCAA	scaffold46
WSSR_73	ATCATCAATCCCAGTCAAAG	AGAAATTCTGGGCTTAGCTT	scaffold48
WSSR_74	TAATTATTGACAGGAGGCGT	GCGAATGACTAGGAGAAATG	scaffold50
WSSR_75	ATCAAATTTGGTCCTCAAAC	GTTGAATGATAATGAAATTGAAA	scaffold51
WSSR_76	AAAGTGAATTCCAGGAAAGT	TCAAATTTGGTCCTCATTCT	scaffold54
WSSR_77	TCCAACACAATCAATCAAGA	TGTAATGAAACCGAGGAATC	scaffold56
WSSR_78	GAGGTCCAGTGAATTACAGC	ATAAAGGAGGAGGAGACTGG	scaffold56
WSSR_79	CTATCTTGAAGTCCCTGTGC	AAAGCACTCTTTCTTTCCCT	scaffold56
WSSR_80	TCTCTTATCACACCACGACA	ATACAGATCGGAGATGCCTA	scaffold56
WSSR_81	CAGCTTGCAGCATCTACATA	CAATTTAAACTCGCCAAATC	scaffold62
WSSR_82	AAACCCACTCCATAAATCAA	TCCGATTCAACTCAGTTACC	scaffold62
WSSR_83	GCTCGACGTTAGAGAGAGAA	TCTCAATCTACCAAGCCCTA	scaffold62

Primer name	Forward primer (5'-3')	Reverse primer (5'-3')	Scaffold
WSSR_84	ACAAATCACAGAGACCAAGG	ATCTCTCTCACTTTGCCGTA	scaffold63
WSSR_85	ATTTAATTTCTCAGCAGCCA	AAACTCACCACCATTGTAGC	scaffold63
WSSR_86	GCATTCATCTCATAATTT	ATTCTTGATGTTCGTATT	scaffold64
WSSR_87	GATAACAACAAGATGGGTC	AACTGTAAATGAGCGGTA	scaffold64
WSSR_88	CACAAATCTTATTGGAAAAC	TTACTACTGATGCTGTTC	scaffold64
WSSR_89	TTGGCAGTTATGTCTCCA	AGTTTGTCCAAGTGTCCC	scaffold64
WSSR_90	GGACGAATCCAACCTGAC	CCTGCTTGCCTGAATCTC	scaffold64
WSSR_91	CATCGTGCCCAGTAAGGA	ACATAGGAAGCGGGTGGT	scaffold64
WSSR_92	TTGCTTATCATAATTCTTGG	CTCTTTTGGGTTTCCTAC	scaffold64
WSSR_93	CTCGTCTGTCCCTATCTT	TGGAGACTACAGCGTTAA	scaffold64
WSSR_94	ACAAGGCATCAAAGTAGCA	CTCCAGGAGATCCAAGACG	scaffold64
WSSR_95	TATTATGCAAAACTATGC	ATAACCTATGTGATTGGG	scaffold64
WSSR_96	CAGAGCAATCAAAGAATA	TCTAAAGCAAGGTTGTCA	scaffold64
WSSR_97	GCGAGTTTGTTTTAGGAG	TTGACATGAATTCTGCAC	scaffold64
WSSR_98	GTCTGAACCCTCATCTAT	CTGGAATCCATAATACAC	scaffold64
WSSR_99	TCACTTGCCGCCCTTCTT	TGACGCCGCTGTAACCAC	scaffold64
WSSR_100	GCAAAAGCCAAAAGGAGA	AACCAGCAGAGGAAAGTG	scaffold64
WSSR_101	AAAAGGTTATTTGATACACG	CATTCCACTAAAGACATTGA	scaffold64
WSSR_102	TAGTGGTTCAACCTTCTT	AACATTGGTTTCTTGATT	scaffold64
WSSR_103	TTTATCGGAGAACTGGAAGA	CTACAAGAAACCAAACACCC	scaffold67
WSSR_104	CAAATTTGATCCTCATTCTTT	TCCAGTGTTTGGTTATGTCA	scaffold67
WSSR_105	TAATGGTGGGATAGAAGTGG	TTTCCTGTGGTCTATTTGCT	scaffold67
WSSR_106	GAATGGGAATGAAATTGAAA	TTTATCAAATTTGGTCCTCG	scaffold68
WSSR_107	AACAGGAGAGCAGTCTCAAA	ACATTAAATGCCCACTGAAC	scaffold68
WSSR_108	ACACATCCTCATTCATTGGT	CAAGAACACCAGACTCCCTA	scaffold69
WSSR_109	AAAGAATATGTCATGTGCCC	TAGGGTGTCATCCTTGTCTC	scaffold69
WSSR_110	AAAGCTCTCTTGGTGGTACA	TGGGAAAGAACTTGAAGAGA	scaffold70
WSSR_111	CCAAATTGATCAAGCTCTTC	TCCAACACATTTGATCTTCA	scaffold71
WSSR_112	CATCTTCCTTGAACCTCTTG	GAAAGATGCAATTCTTGAGG	scaffold72
WSSR_113	AGTCGTTTACACGCTTGATT	TGAGGAAGAAGATCCAAAGA	scaffold72
WSSR_114	GATAGATCACACCAAGGCAT	CTGTTCCTCAAACCAAAGAG	scaffold72
WSSR_115	CAGGAAGGTGAAGAAGAGAA	TACATACTCGCAGTGCAGAC	scaffold74
WSSR_116	CTCGTAGAAGAGCTTCCTGA	AACAAGCTAGCAGAGATTGC	scaffold75
WSSR_117	ATCAAATTTGGTCCTCAAAC	AAAGGTTGAATGAGAATGAA	scaffold76
WSSR_118	TAGTCTCAGAATCGGTGAGG	CCCAACCAATACAAACATTC	scaffold76
WSSR_119	TATTTCGCATCACAAACAAG	GTGAATGGTGAGGGTAGAAA	scaffold77
WSSR_120	GTTCAGATCCTGAAGCAAAG	ATGTGAGGTGAAAGTATGGC	scaffold88
WSSR_121	GGTTACCAAATTTGCATGAT	ATTTGCATTTCCAAGATCAC	scaffold94
WSSR_122	TTCTTAAGCTTTGAGATGCC	ATGCTTGTGCATGTGTGTAT	scaffold100
WSSR_123	GGATACTGTTGCTTCTGGAG	AATGGCTTGCTTGTAAATGT	scaffold102
WSSR_124	TGCTCTGAAAGATCTACGGT	AACCACATTGATTCTTCCAC	scaffold103
WSSR_125	GCACTTTCCAACAGGTATGT	AAATCAATTGCCTGGATAAA	scaffold105

Primer name	Forward primer (5'-3')	Reverse primer (5'-3')	Scaffold
WSSR_126	GGGATCAAACAATCACTACAA	TTAAATTACACTTGGTGCCC	scaffold105
WSSR_127	AAGCTTTACCAAATCACCAA	TGTTGGCACAATCTCATCTA	scaffold106
WSSR_128	TTTATGCTGTAGGAGTCGGT	TGATATCGCTAACTGCAATG	scaffold111
WSSR_129	ACTAACACACCCTCCTCCTT	AGTAAACAGCATGAGCAGGT	scaffold111
WSSR_130	CCATAGATTTAGGCTCTGGA	GGATCTCATCTTGTCTGCAT	scaffold113
WSSR_131	GGGACGTATAGGAGGAGAAC	TGTTTCTAGGGTGAATGCTT	scaffold116
WSSR_132	CAACAAGAGTTGAGAGGAGG	TTTGGAACATTCTCAACCAT	scaffold118
WSSR_133	AAGACGAAGAGAGAGAGCCT	CTTCCTCAACATCATCCACT	scaffold120
WSSR_134	ACCTGGGTAATAAGATTGGG	ATCATTAGAGCCACATCCAC	scaffold126
WSSR_135	GATCCATTGAATTCACCATC	CAGCTCTTCTGGTTAAGGAA	scaffold138
WSSR_136	GATCAAGGTCACGCTCTAAG	GTACAACCCATTCAGAGACC	scaffold140
WSSR_137	ACAGACCCACTGTTATCGAC	ATTCCTACCTCTAAATGGGC	scaffold145
WSSR_138	TGAGAAGTGAGTTGTGTCCA	ATGACTGATGCTTTCAATCC	scaffold146
WSSR_139	ATAAGGTGGAAGATGGGATT	CAAATCTGCATTAGCAAACA	scaffold146
WSSR_140	TCACCATCTTCCATCTTCTC	GGTGAGAGAGACCCTTTCTT	scaffold157
WSSR_141	TACTACATTCATGCAGCCAA	TGAGGTGTCCACTGTTAGGT	scaffold157
WSSR_142	GTCCAAGATAATTGCAGCTC	ATTCCCAGCAACTTGTTAAA	scaffold157
WSSR_143	TTTGGGTACAAATCGCTAAT	TATTCCTCCTCCTCCTCTTC	scaffold159
WSSR_144	ATCTGATCCACAACATCCAT	ACATTCTGTGACATGGGAAT	scaffold159
WSSR_145	TTGTTCTGATGCCTTTACCT	TTTGCAGTAGTGAGAAGCAA	scaffold161
WSSR_146	TAACCATACATGCACACGTT	CCATTGGATTCTGAGATTGT	scaffold169
WSSR_147	TTGACTGTGCAGATGTTGAT	AAATATGACCCAAACCACAG	scaffold174
WSSR_148	AATTGTGGAGCTTGGTTAAA	AAGGTTGTTCATTTGATTGG	scaffold176
WSSR_149	GCTGTAAATATGAGGAACCG	AATTTGAGTGGATGAATGGA	scaffold176
WSSR_150	TTTGAGACCATGGAACTACC	TGAAAGCACAAACTTCACAG	scaffold177
WSSR_151	TTGCTTCAACCAGTAACCTT	ATAGCTGGAGCTCCTTTCTT	scaffold177
WSSR_152	ATAAATCTGGGACCACTCCT	AAACCCATAATTAAACGCAA	scaffold189
WSSR_153	ACTCCGATTTCAATAGCTCA	ATTAAAGCCAAACGTACCAA	scaffold196
WSSR_154	GGAAGAGGATGGAAAGAACT	CACAGCTAAACATGCTTGAA	scaffold199
WSSR_155	CAGGAACTTCTTTACAGCGT	AATAGCTGAACTATGCCGAG	scaffold203
WSSR_156	TTTCAATTTCATCCCTTAGAA	AATGAGGAACAAATCTTACAAA	scaffold206
WSSR_157	GAATCCCAGGTTTAGTACCC	AGGATTCCAGAGTCTCTTCC	scaffold206
WSSR_158	CTGCCCTCTGACGACTTACT	TTATCTCGGCTTATCCTCCC	scaffold207
WSSR_159	CGAGGAGAAGATTAGAGTGG	TTGACGGAAAGGATTGAGTT	scaffold207
WSSR_160	AGTGGGTGTTTAGCATTGTG	GAGATTCTTGTCAGCGGGAG	scaffold207
WSSR_161	GATTTGTGGTGGTAGTAGATT	TTTGTTAGACTGTTGGGATA	scaffold207
WSSR_162	CCATGCACATACCCTAATCT	ACAAGGTTATCCATGCAGAC	scaffold208
WSSR_163	GGAACTTGTATTCTCACCCA	GTGAGTTCTTGAACCAAAGC	scaffold212
WSSR_164	TCTTACATCTGCATTGGTGA	ATCTACCGTGCTTCCTCATA	scaffold215
WSSR_165	CCTATTCACTCGAAACTCTT	CTGTATGGTCTTATCTGGGT	scaffold238
WSSR_166	GGGTAGGGTACGAGAAAGA	GGAGGAGCAGGAATCAATA	scaffold238
WSSR_167	AAGGAGGCAGCGAGGAAGT	TTGGGAATGCGAGGAGAAC	scaffold238

Primer name	Forward primer (5'-3')	Reverse primer (5'-3')	Scaffold
WSSR_168	TATGCCACAAGATATGAGCA	ATATAGCTTGCCCAGTCAAA	scaffold242
WSSR_169	AACCTTGGCCTAGTCTCTCT	ACTTGACCATATGCATGAAA	scaffold251
WSSR_170	AAGCAGTGACAGCGGTAG	TACTTCGCGTCAGGTTTT	scaffold253
WSSR_171	ATCAAATCACGCTAATCC	AACAAGAAAGCAACATCG	scaffold253
WSSR_172	CCCACCAAAGCGTCTGTC	CGAGTTGTTGGGCTGGAT	scaffold253
WSSR_173	TTATTGCTGGAAAGGTTG	TTCGTGTCTTTAGGGTCT	scaffold253
WSSR_174	TGGGTCCATTCTCAACTA	AAGGGACTCAGCAAAGAT	scaffold253
WSSR_175	GGGAAGATCGCCTGTTTA	TGACAGCGTTGCGTTTGA	scaffold253
WSSR_176	ACCCAAGAAGACAAGACAC	TATCACTAACAAGCCCATC	scaffold253
WSSR_177	AACATTCTGCTTCTTCCTTT	AACCTCCATTACCATCCATA	scaffold254
WSSR_178	CACTGCTATTTCCCTTGTTA	TCTATCTTTCTCGGTTGGTA	scaffold254
WSSR_179	ATTCCACAGAAGCAATCATC	AGGAAGGAGATATTGAAGGC	scaffold259
WSSR_180	ATCGTCCTCCTTCTTGGTG	AACAGAACCTGGGATTTGC	scaffold260
WSSR_181	AACTTGAAGGGTAGGACTGC	TAAAGGGAAGATGCTAACTC	scaffold260
WSSR_182	CTCCCAGTTTGTTTCCATC	TAGAGGTGCTTGCTTCAGT	scaffold265
WSSR_183	TCTCCCTTTCTCACCTCT	AACTCCTACCCACTTTCG	scaffold265
WSSR_184	TGGGAGGAGTGTCAGAAG	CTCCATAACAACCAGCAA	scaffold265
WSSR_185	AGTTGGAAATAGTTGGCGTA	GATGAGCGTGTGACTGTAAA	scaffold268
WSSR_186	CAGGAGCCATAGCTTTCTTA	ATCTGGTTCCTGATGAGTTG	scaffold290
WSSR_187	AAAGAAGGCAAACAAAGCA	AAACAGCGAAAGAAGCAAA	scaffold292
WSSR_188	AATGACTGTATTGTGGCTTCT	TGATTGGGATTTACGACTG	scaffold292
WSSR_189	CTTGGGATAACTGCTGGAA	TGTGGAAGATGATGGGAG	scaffold292
WSSR_190	TGTTCATCATTGCCTTGT	TACTGTCTTTGCCACCAT	scaffold292
WSSR_191	AGGTGTAGAAAACCTCAG	ATCATTCAAAGCCAGTGT	scaffold452
WSSR_192	GGAATATGATGAGTGAAT	AAGTTATACCCTATGAGA	scaffold452

Table S2 Allele number at each locus generated by 10 SSR primers in the four willow species

Primer name / Sample name	WSSR_11	WSSR_33	WSSR_34	WSSR_88	WSSR_89	WSSR_91	WSSR_94	WSSR_100	WSSR_124	WSSR_173
**Sba_1**	2	3	1	1	2	2	3	4	1	1
**Sba_2**	2	3	1	1	2	2	3	4	1	1
**Sba_4**	2	3	1	1	2	2	3	4	1	1
**Sba_5**	2	2	2	1	2	2	3	4	1	1
**Sba_7**	2	2	2	1	2	2	3	4	1	1
**Sba_9**	2	2	2	1	2	2	3	4	1	1
**Sba_10**	2	3	1	3	3	2	3	4	1	1
**Sba_11**	2	3	1	3	3	2	3	4	1	1
**Sba_13**	2	2	2	2	2	2	3	4	1	2
**Sba_14**	2	2	2	2	2	2	3	4	1	2
**Sba_15**	2	1	2	2	3	2	4	4	2	1
**Sba_17**	2	1	2	2	3	2	4	4	2	1
**Sma_1**	2	1	2	4	2	2	4	4	2	1
**Sma_2**	1	1	2	0	1	1	1	1	1	0
**Sma_3**	2	1	2	4	2	2	4	4	2	1
**Sma_5**	2	1	2	4	2	2	4	4	2	1
**Sma_6**	2	1	2	1	3	2	3	4	2	2
**Sma_7**	1	1	1	2	1	1	2	2	1	1
**Sma_9**	2	1	2	1	3	2	3	4	2	2
**Sma_11**	1	1	1	2	1	1	2	2	1	1
**Sma_16**	2	2	1	1	2	2	2	2	1	1
**Sma_18**	2	2	2	1	2	2	3	4	1	2
**Sma_21**	2	2	2	1	2	2	3	4	1	2
**Sma_26**	2	2	2	1	2	2	3	4	1	2
**Sin_47**	1	2	2	1	2	0	2	2	1	2
**Sin_74**	1	2	2	1	2	2	2	2	2	2
**Sin_99**	1	2	2	1	2	2	2	2	2	2
**Sin_134**	1	2	2	1	2	0	2	1	2	2
**Sin_137**	1	2	2	1	2	2	2	1	2	2
**Sin_221**	1	2	2	1	2	2	2	1	2	2
**Sin_270**	3	4	3	1	4	2	2	4	3	4
**Sin_491**	2	2	1	1	2	2	2	1	2	2
**Sin_551**	1	2	1	2	1	2	2	2	2	2
**Sin_578**	1	2	2	1	2	1	2	1	2	2
**Sin_579**	1	2	2	1	2	1	2	1	2	2
**Sin_608**	1	2	2	1	1	1	2	2	0	1
**Ssu_1**	2	2	1	2	2	1	2	2	2	2
**Ssu_2**	1	1	1	2	2	1	2	2	2	2
**Ssu_17**	2	2	2	2	2	1	2	2	1	2
**Ssu_38**	2	2	2	2	2	1	2	2	1	2
**Ssu_47**	2	1	2	1	1	1	1	2	1	2

Primer name / Sample name	WSSR_11	WSSR_33	WSSR_34	WSSR_88	WSSR_89	WSSR_91	WSSR_94	WSSR_100	WSSR_124	WSSR_173
**Ssu_50**	2	2	2	2	2	2	1	2	2	2
**Ssu_69**	1	2	2	2	2	2	1	2	2	2
**Ssu_90**	1	2	3	2	2	3	3	3	1	2
**Ssu_99**	2	1	2	1	1	1	2	2	2	2
**Ssu_101**	2	1	2	1	1	1	2	2	0	2
**Ssu_107**	2	2	2	2	2	2	2	2	0	2
**Ssu_120**	1	2	1	2	2	1	1	2	2	2

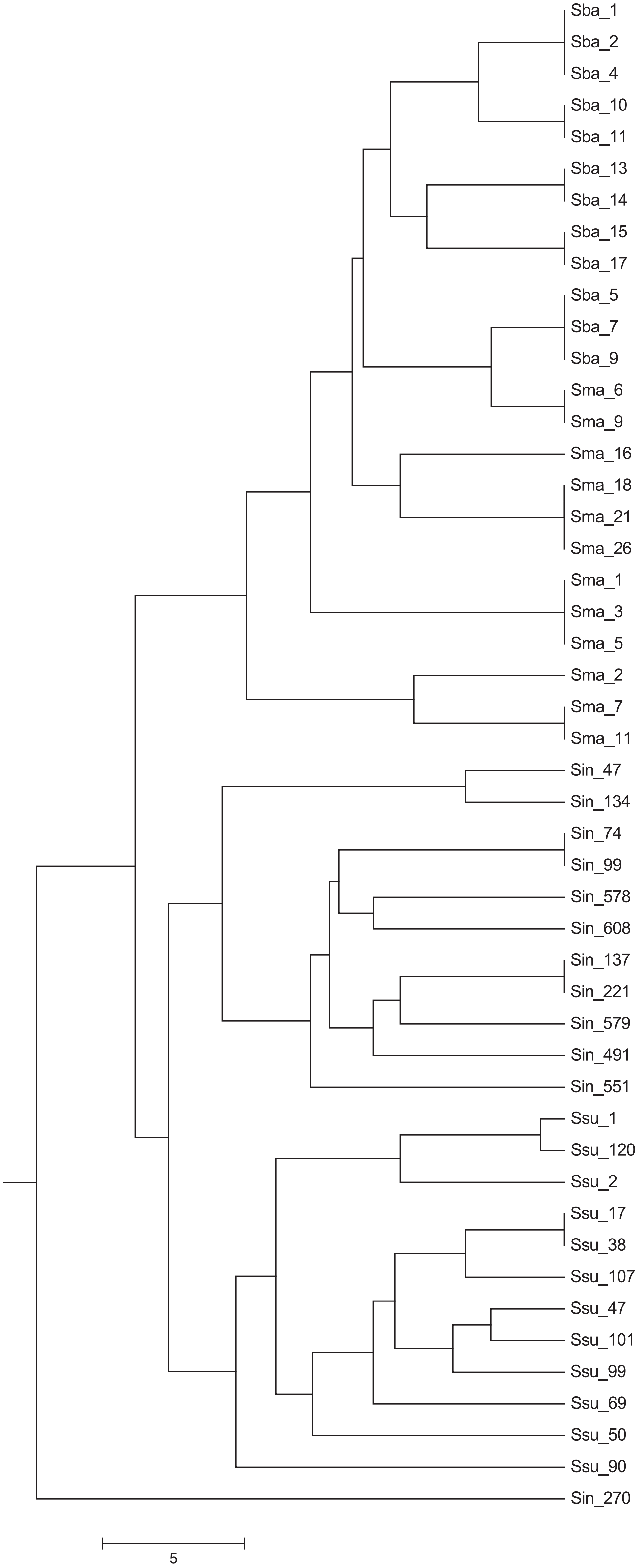
Sba_1
Sba_2
Sba_4
Sba_10
Sba_11
Sba_13
Sba_14
Sba_15
Sba_17
Sba_5
Sba_7
Sba_9
Sma_6
Sma_9
Sma_16
Sma_18
Sma_21
Sma_26
Sma_1
Sma_3
Sma_5
Sma_2
Sma_7
Sma_11
Sin_47
Sin_134
Sin_74
Sin_99
Sin_578
Sin_608
Sin_137
Sin_221
Sin_579
Sin_491
Sin_551
Ssu_1
Ssu_120
Ssu_2
Ssu_17
Ssu_38
Ssu_107
Ssu_47
Ssu_101
Ssu_99
Ssu_69
Ssu_50
Ssu_90
Sin_270
5
